# Quantification of calreticulin redistribution in LNCaP prostate cancer cells using a fixed-cell imaging workflow

**DOI:** 10.17912/micropub.biology.002112

**Published:** 2026-09-15

**Authors:** Janiyah Vogle, Kandace Rankin, Courtney Thomas

**Affiliations:** 1 Biological and Physical Sciences, South Carolina State University, Orangeburg, SC, US

## Abstract

Calreticulin (CRT) is an endoplasmic reticulum chaperone that can localize to the cell surface under stress, where it functions as a damage-associated molecular pattern. Quantifying surface-exposed CRT in prostate cancer models is challenging due to low baseline expression and variability in imaging workflows. We describe a fixed-cell immunofluorescence method to distinguish intracellular and surface CRT in LNCaP cells. Cells treated with thapsigargin exhibited increased surface localization. We introduce a redistribution factor (RF = ectoCRT/(ectoCRT + endoCRT)) to quantify CRT partitioning. This approach provides a reproducible framework for assessing CRT redistribution using standard fluorescence microscopy.

**Figure 1. Validation and robustness of a quantitative Redistribution Factor for measuring calreticulin cell-surface partitioning:** **(A)** The Redistribution Factor (RF), defined as ectoCRT/(ectoCRT + endoCRT) quantifies the fraction of CRT localized to the cell surface, normalizing for the large intracellular pool of calreticulin. Surface GFP-tagged CRT analysis included approximately 23 cells per condition across three independent experiments, while intracellular GFP-tagged CRT analysis included approximately 99 cells per condition across three independent experiments. Each data point represents an individual cell. Fluorescence intensity values were obtained using automated image segmentation under identical imaging conditions. **(B)** Comparison of treatment-induced redistribution factor (RF) between endogenous CRT and GFP-CRT workflows. Both approaches demonstrated similar treatment-dependent increases in RF, with parallel trends across TG and TSA conditions. The close concordance between endogenous and GFP-tagged CRT supports that the GFP reporter successfully recapitulates treatment-induced CRT redistribution, validating the GFP-CRT imaging workflow for quantitative analysis. Endogenous surface calreticulin analysis included approximately 30 cells per condition across 3 independent experiments, while endogenous intracellular CRT analysis included approximately 50 cells per condition across three independent experiments. **(C)** Quantification of endogenous surface-exposed calreticulin (ectoCRT) under non-permeabilized conditions. Parental LNCaP cells were treated with vehicle (0.1% DMSO), thapsigargin (TG), or trichostatin A (TSA). Cells were incubated with anti-CRT antibody before fixation to detect surface-accessible endogenous CRT. Mean fluorescence intensity (arbitrary units, a.u.) increased following TG and TSA treatment, indicating enhanced surface exposure of endogenous CRT. Statistical comparisons between groups are indicated (P values calculated using unpaired T-Test). Surface and intracellular fluorescence were quantified by automated image segmentation under identical image acquisition and analysis parameters for all treatment groups. Endogenous surface calreticulin analysis included approximately 30 cells per condition across 3 independent experiments. **(D)** Quantification of surface-exposed CRT in LNCaP cells expressing GFP-tagged wild-type calreticulin (pMSCV-IRES-GFP/CALR wt). Under identical treatment conditions, TG and TSA increased cell-surface CRT fluorescence compared with vehicle-treated controls. Mean fluorescence intensity (arbitrary units, a.u.) increased following TG and TSA treatment, indicating enhanced surface exposure of GFP-tagged CRT. Statistical comparisons between groups are indicated (P values calculated using unpaired T-Test). Surface GFP-tagged CRT analysis included approximately 23 cells per condition across three independent experiments. **(E)** Quantification of endogenous intracellular calreticulin following permeabilization. Cells were permeabilized with 1% Triton X-100 prior to immunostaining to measure intracellular CRT. Mean fluorescence intensity decreased following TG and TSA treatment relative to vehicle-treated cells, consistent with redistribution of CRT from intracellular compartments to the plasma membrane. Statistical comparisons between groups are indicated (P values calculated using unpaired T-Test). Endogenous intracellular CRT analysis included approximately 50 cells per condition across three independent experiments. **(F)** Quantification of intracellular GFP-CRT following permeabilization. Intracellular fluorescence decreased after TG and TSA treatment, mirroring the redistribution observed for endogenous CRT. Intracellular GFP-tagged CRT analysis included approximately 99 cells per condition across three independent experiments. **(G)** Representative immunofluorescence images of endogenous CRT. The left panels show surface CRT staining, while lower panels show intracellular CRT detected under permeabilized conditions. Images illustrate reduced intracellular CRT and increased surface CRT following TG and TSA treatment. Scale bars = 10 μm. **(H)** Comparison of redistribution factor (RF) between an exposure time of 250ms and 1000ms. Both exposures showed strong agreement across a four-fold exposure range indicating the RF is largely independent of exposure settings in this large range, supporting its robustness across acquisition settings. **(I)** Schematic illustrating calreticulin redistribution during immunogenic cell death. Under baseline conditions CRT is predominantly retained within the endoplasmic reticulum. ER stress induces CRT translocation to the plasma membrane while a fraction remains intracellular. The redistribution factor (RF) provides quantitative value for the proportion of total detectable CRT localized to the cell surface.

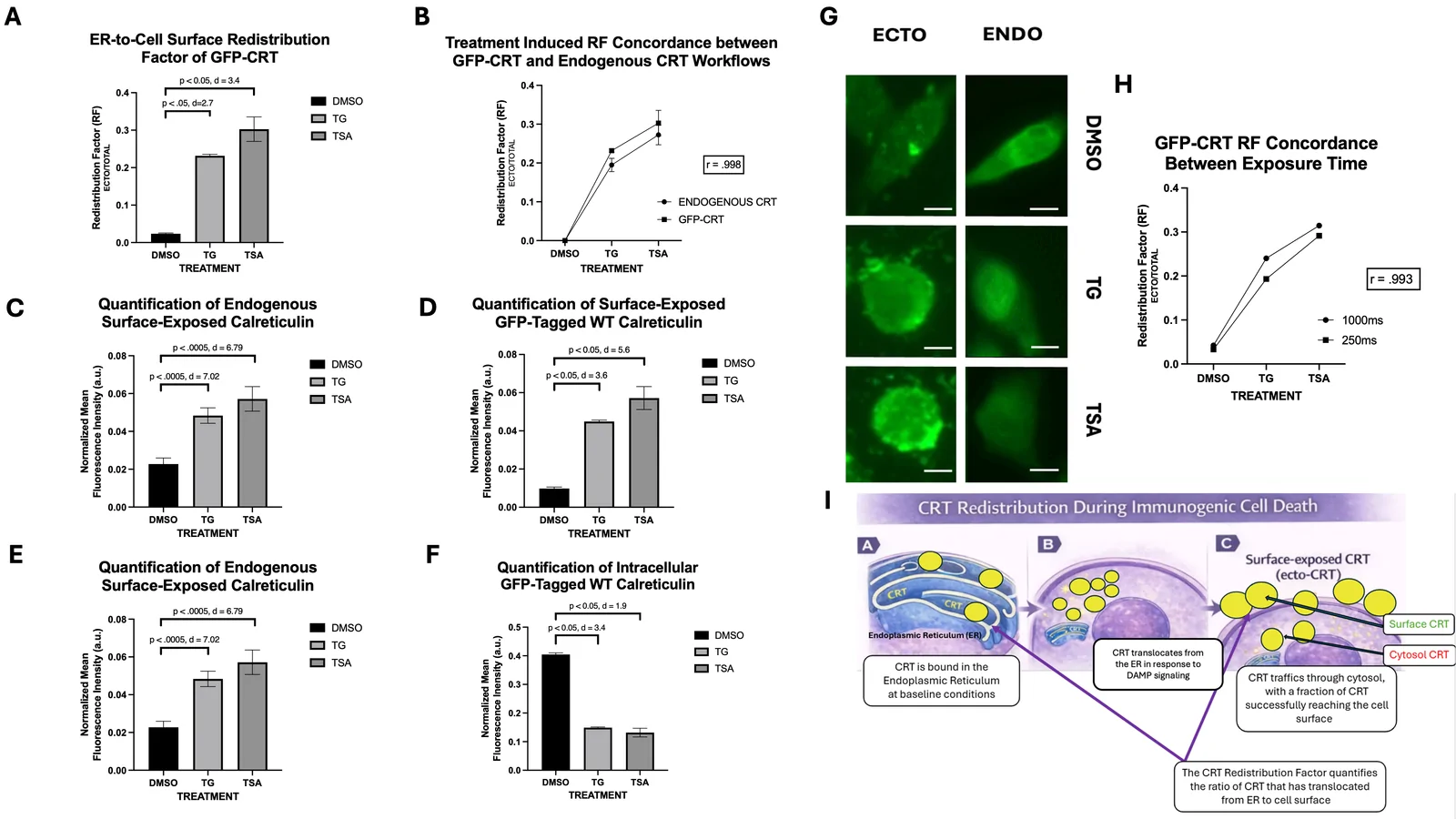



## Description

Calreticulin (CRT) is a 46 kDa endoplasmic reticulum (ER)–resident chaperone protein that plays a critical role in calcium homeostasis and glycoprotein folding (Michalak et al., 1999). In addition to these canonical ER functions, CRT can become detectable at the plasma membrane under conditions of cellular stress, where it functions as a damage-associated molecular pattern (DAMP) involved in immune recognition and clearance of stressed or damaged cells (Gardai et al., 2005; Obeid et al., 2007). Surface-exposed CRT has been described in several malignancies, including bladder, lung, breast, and ovarian cancers, where it has been associated with altered cellular signaling and immune engagement (Kageyama et al., 2004; Liu et al., 2012; Song et al., 2012). In prostate cancer, however, reports of CRT expression and localization have been inconsistent, with both increased and decreased expression described depending on disease context (Alur et al., 2009). These discrepancies highlight the need for reproducible and quantitative approaches to evaluate CRT localization in prostate cancer models.

CRT contains three structural domains (N, P, and C), including a C-terminal Lys-Asp-Glu-Leu (KDEL) sequence that mediates ER retention through receptor-dependent retrieval pathways (Cabrera et al., 2003; Michalak et al., 1999). Despite this retention mechanism, CRT has been observed at the cell surface under conditions of cellular stress, suggesting that redistribution from the ER can occur. The processes governing CRT redistribution remain incompletely defined and may involve multiple cellular pathways, including ER stress responses and post-translational modifications. Proteomic studies have demonstrated that ER-resident proteins can undergo lysine acetylation (Pehar et al., 2012), and altered acetylation profiles have been observed in prostate cancer cells (Pathak et al., 2015). These findings provide biological context for evaluating CRT localization under pharmacologic conditions that influence cellular stress and acetylation state, although the present study does not directly assess these mechanisms.

Quantification of surface-exposed CRT (ectoCRT) in prostate cancer models presents several technical challenges. LNCaP cells exhibit relatively low baseline surface CRT expression, making detection sensitive to staining conditions and imaging parameters. Variability in fluorescence intensity measurements can arise from differences in antibody accessibility, imaging exposure, and segmentation approaches. Furthermore, many studies of protein trafficking rely on live-cell imaging or time-lapse microscopy, which may not be accessible in all research environments. These limitations underscore the need for a standardized, reproducible fixed-cell imaging workflow that enables reliable distinction between intracellular and surface-accessible CRT pools.

In this study, we optimized a non-permeabilized immunofluorescence protocol to detect surface CRT in LNCaP cells using total anti-CRT antibody staining, with complementary experiments performed in cells transfected with wild-type CRT from the pMSCV-IRES-GFP/CALR wt plasmid. Surface staining was performed by incubating cells with primary antibody prior to fixation, restricting antibody access to extracellular epitopes. Intracellular CRT detection required permeabilization using 1% Triton X-100 in phosphate-buffered saline (PBS). Membrane integrity under non-permeabilized conditions was validated using ERp57 as an intracellular control protein, which showed no detectable signal in the absence of permeabilization, confirming that intracellular epitopes remained inaccessible during surface staining.

Cells were treated with thapsigargin (TG), a well-established ER stress inducer known to influence CRT cell surface translocation (Jeffery et al., 2011), as a positive control condition. Comparative treatment with trichostatin A (TSA), a potent histone deacetylase (HDAC) inhibitor (Frønsdal et al., 2004), was used to evaluate increased acetylation conditions on CRT trafficking. Given that altered acetylation profiles are observed in prostate cancer (Watson, et al., 2010), we utilized TSA to test whether increased global acetylation influences CRT surface mobilization, serving as a secondary stress-response model alongside TG. 0.1% DMSO was included as a vehicle control. TG and TSA concentrations were determined by preliminary Methyl Thiazolyl Tetrazolium (MTT) optimization assays and supporting literature on ER stress induction and HDAC inhibition in prostate cancer models. Conditions were chosen that produced detectable CRT redistribution while not compromising cell integrity. Under TG treatment conditions, a visible redistribution of CRT signal toward the cell periphery was observed relative to vehicle-treated controls. While intracellular CRT fluorescence intensity decreased and surface-associated signal increased, direct comparison of these measures revealed weak correlation across treatment conditions. These findings indicate that absolute fluorescence intensity measurements alone may not fully capture the extent of CRT redistribution.

To address this limitation, we implemented a redistribution factor (RF) defined as ectoCRT/(ectoCRT + endoCRT), which quantifies the relative partitioning of CRT between intracellular and surface compartments within individual cells. By normalizing surface-associated signal to total detectable CRT, RF provides a metric that is less influenced by global changes in protein expression or imaging variability. This approach is particularly useful in systems with low baseline surface expression, where small changes in fluorescence intensity may be difficult to interpret using absolute measurements alone. Across independent experiments and standardized imaging conditions, RF values demonstrated reproducibility and sensitivity to treatment-dependent changes in CRT localization.

Importantly, the present study does not directly evaluate the molecular mechanisms underlying CRT redistribution but instead establishes a reproducible imaging and quantification framework for assessing CRT partitioning in prostate cancer cells. This fixed-cell approach eliminates the requirement for live-cell imaging systems and enables broader application in laboratories equipped with standard fluorescence microscopy platforms.

In summary, we describe a standardized non-permeabilized immunofluorescence workflow and a redistribution-based quantification strategy for evaluating ER-to-surface CRT mobilization in LNCaP prostate cancer cells. This method provides a practical and reproducible approach for measuring CRT redistribution and may facilitate future studies investigating protein trafficking, cellular stress responses, and DAMP-associated signaling in cancer models.

## Methods

Cell Culture and Transfection

LNCaP human prostate adenocarcinoma cells were maintained in RPMI-1640 medium supplemented with 10% fetal bovine serum and 1% penicillin–streptomycin under standard culture conditions (37°C, 5% CO₂). For transfection studies, cells were transiently transfected with GFP-tagged calreticulin (pMSCV-IRES-GFP/CALR wt plasmid - a gift from Norio Komatsu) and incubated for 24 hours prior to treatment. GFP fluorescence was used as the primary readout for total CRT expression across all experimental conditions.

Drug Treatments

Following drug administration, cells were incubated for 4 hours for endogenous CRT experiments or 24 hours for transfected (GFP-CRT) experiments with:

- Vehicle control (0.1% DMSO)
- Thapsigargin (TG; 100 nM)
- Trichostatin A (TSA; 50 nM)

These conditions were selected to evaluate CRT redistribution under pharmacologic perturbation.

Immunofluorescence Staining

Surface CRT (ectoCRT) Staining:

Surface-accessible CRT was detected under non-permeabilized conditions. Cells were incubated with either anti-CRT (for endogenous CRT experiments) or anti-GFP (for transfection experiments) primary antibody prior to fixation to restrict antibody access to extracellular epitopes. Following primary antibody incubation, cells were fixed with 2% paraformaldehyde (PFA) and subsequently incubated with fluorophore-conjugated secondary antibodies for 1 hour at room temperature. Nuclei were stained with DAPI.

Membrane integrity was validated using ERp57 as an intracellular control protein, which showed no detectable signal under non-permeabilized conditions, confirming that intracellular epitopes remained inaccessible.

Intracellular CRT Staining:

For intracellular CRT detection, cells were fixed with 4% paraformaldehyde (PFA), washed with 0.01 M phosphate-buffered saline (PBS), and permeabilized using 1% Triton X-100 in PBS. Cells were then blocked in normal donkey serum and incubated with either anti-CRT or anti-GFP primary antibody overnight at 4°C. Following washing, cells were incubated with fluorophore-conjugated secondary antibodies and counterstained with DAPI.

Image Acquisition

Fluorescence images were acquired using identical imaging settings across all treatment conditions to ensure comparability. Exposure time was fixed at 1 ms for all channels and samples to minimize variability in fluorescence intensity measurements.

Image Analysis and Quantification

Image analysis was performed using CellProfiler on 16 bit images with custom pipelines designed for single-cell segmentation and fluorescence quantification.

- Intracellular CRT was quantified using expanded DAPI-based nuclear masks to capture cytoplasmic signal.
- Surface CRT was quantified using CRT-based segmentation to detect membrane-associated fluorescence.

Background fluorescence was quantified from cell-free regions outside segmented cell regions of interest (ROIs) within each image. The average background fluorescence intensity was subtracted from the mean fluorescence intensity of each ROI to generate corrected CRT intensity measurements. Area-based and intensity-based filtering were then applied to exclude debris and incorrectly segmented objects before calculation of fluorescence intensity and redistribution factor (RF) values.

For endogenous experiments, approximately 30 cells were analyzed for surface CRT and 50 cells for intracellular CRT per condition. For GFP-transfected experiments, approximately 23 cells were analyzed for surface CRT and 99 cells for intracellular CRT per condition. All analyses were performed across three independent experiments.

Experimental conditions were validated in parallel using both endogenous staining (anti-CRT) and transfected reporters (anti-GFP) to confirm consistency. 

Use of a single GFP-CRT reporter for transfection experiments across all conditions minimized inter-channel variability and enabled direct comparison of CRT redistribution between treatments.

Redistribution Factor Calculation

Background-subtracted mean fluorescence intensity values were used to calculate a redistribution factor (RF) for each cell, defined as:

RF= ectoCRT /ectoCRT + endoCRT 

ectoCRT = surface CRT and endoCRT = intracellular CRT

This metric represents the relative partitioning of CRT between surface-accessible and intracellular compartments.

Statistical Analysis

Fluorescence intensity measurements were obtained at the single-cell level for both intracellular and surface conditions. Statistical comparisons between treatment groups were performed using unpaired two-tailed Student’s t-tests.

Comparisons were conducted between vehicle (0.1% DMSO) and treatment conditions (thapsigargin [TG] or trichostatin A [TSA]) for:

- Surface CRT fluorescence intensity
- Intracellular CRT fluorescence intensity
- Redistribution factor (RF)

Sample sizes varied by experiment: for endogenous experiments, approximately 30 cells were analyzed for surface CRT and 50 cells for intracellular CRT per condition. For GFP-transfected experiments, approximately 23 cells were analyzed for surface CRT and 99 cells for intracellular CRT per condition. All analyses were conducted across three independent experiments.

Data are presented as mean ± standard deviation. Statistical significance values (p) and effect sizes (Cohen’s d) are reported in the figure.

## Reagents

**Table:** 

Reagent	Source	Catalog Number
pMSCV-IRES-GFP/CALR wt plasmid	Addgene	plasmid # 214699
GFP antibody	Thermo Fisher Scientific	PIMA515256
CRT antibody	Cell Signaling Technology	12238
ERp57 antibody	Proteintech	15967-1-AP
Thapsigargin	Enzo Life Sciences	NC9006970
Trichostatin A	Thermo Fisher Scientific	502707197
Triton X-100	Millipore Sigma	648466
Dimethyl sulfoxide (DMSO)	Thermo Scientific	D12345
Paraformaldehyde	Thermo Scientific	043368.9L
